# Cutaneous adverse reactions and anti-tumor effects of anti-CCR4 monoclonal antibody (mogamulizumab) on adult T-cell leukemia-lymphoma

**DOI:** 10.1186/1742-4690-12-S1-P66

**Published:** 2015-08-28

**Authors:** Kentro Yonekura, Masahito Tokunaga, Nobuyo Kawakami, Koichiro Takeda, Tamotsu Kanzaki, Yoshifusa Takatsuka, Nobuaki Nakano, Ayumu Kubota, Syogo Takeuchi, Atae Utsunomiya

**Affiliations:** 1 Department of Dermatology, Imamura Bun-in Hospital, Kagoshima, Japan; 2 Department of Hematology, Imamura Bun-in Hospital, Kagoshima, Japan

## 

The novel defucosylated humanized monoclonal antibody against CC chemokine receptor 4 (CCR4), mogamulizumab, exhibited strong effects on adult T-cell leukemia-lymphoma (ATL) in a phase II clinical study. It has been also reported that mogamulizumab frequently developed cutaneous adverse reactions (CAR) and a close association between CAR and the prognosis. [1] We studied the effects of mogamulizmab on ATL and CAR in 32 patients with ATL. Informed consent was obtained prior to study. Nineteen patients received more than 4 cycles of mogamulizumab therapy. Twenty patients developed an infusion reaction, and eight patients suffered from CAR. These eight patients were administered mogamulizumab more than 4 cycles (median: 7 cycles). The severity of CAR according to CTCAE v3.0 was grade 1 in one patient, 2 in two, 3 in three and 4 in two patients respectively. In six of 8 patients, CARs appeared or most worsened in more than 4 weeks after the last administration. All CARs fortunately subsided later. One patient, however, suffered from toxic epidermal necrolysis and other CARs (erythroderma and generalized exudative erythema in two each patients) prolonged for several months. As for anti-tumor effects with more than 4 cycles of treatments, overall response rate (complete remission (CR) and partial remission (PR)) was 58% (CR: 6, progressive disease (PD): 2) with CAR, and 36% (CR:3, PR:1, stable disease (SD):4, PD:3) without CAR. These results suggest that 1) the incidence of CAR were associated with the frequency of mogamulizumab administration, 2) CARs might be favorable signs of the effect, and 3) follow-up with careful attention to CAR was recommended for at least several months after the finish of treatments.

